# Evaluation of bioaerosol samplers for the detection and quantification of influenza virus from artificial aerosols and influenza virus–infected ferrets

**DOI:** 10.1111/irv.12678

**Published:** 2019-09-21

**Authors:** Christian Bekking, Lily Yip, Nicolas Groulx, Nathan Doggett, Mairead Finn, Samira Mubareka

**Affiliations:** ^1^ Department of Laboratory Medicine and Pathobiology Faculty of Medicine University of Toronto Toronto Ontario Canada; ^2^ Biological Sciences Sunnybrook Research Institute Toronto Ontario Canada; ^3^ Present address: Faculty of Medicine Dalhousie University Halifax Nova Scotia Canada; ^4^ Present address: Department of Microbiology University of Waterford Waterford Ireland

**Keywords:** bioaerosol samplers, bioaerosols, ferret model, influenza virus

## Abstract

**Background:**

Bioaerosol sampling devices are necessary for the characterization of infectious bioaerosols emitted by naturally‐infected hosts with acute respiratory virus infections. Assessment of these devices under multiple experimental conditions will provide insight for device use.

**Objectives:**

The primary objective of this study was to assess and compare bioaerosol sampling devices using a) an in vitro, environmentally‐controlled artificial bioaerosol system at a range of different RH conditions and b) an in vivo bioaerosol system of influenza virus‐infected ferrets under controlled environmental conditions. Secondarily, we also sought to examine the impact of NSAIDs on bioaerosol emission in influenza virus‐infected ferrets to address its potential as a determinant of bioaerosol emission.

**Methods:**

We examined the performance of low and moderate volume bioaerosol samplers for the collection of viral RNA and infectious influenza virus in vitroand in vivo using artificial bioaerosols and the ferret model of influenza virus infection. The following samplers were tested: the polytetrafluoroethylene filter (PTFE filter), the 2‐stage National Institute of Occupational Safety and Health cyclone sampler (NIOSH cyclone sampler) and the 6‐stage viable Andersen impactor (Andersen impactor).

**Results:**

The PTFE filter and NIOSH cyclone sampler collected similar amounts of viral RNA and infectious virus from artificially‐generated aerosols under a range of relative humidities (RH). Using the ferret model, the PTFE filter, NIOSH cyclone sampler and the Andersen impactor collected up to 3.66 log_10_copies of RNA/L air, 3.84 log_10_copies of RNA/L air and 6.09 log_10_copies of RNA/L air respectively at peak recovery. Infectious virus was recovered from the PTFE filter and NIOSH cyclone samplers on the peak day of viral RNA recovery.

**Conclusion:**

The PTFE filter and NIOSH cyclone sampler are useful for influenza virus RNA and infectious virus collection and may be considered for clinical and environmental settings.

## 1 INTRODUCTION

Influenza virus remains a public health concern due to associated seasonal burden of disease and pandemic potential.^1^, ^2^ Person‐to‐person transmission occurs by direct and indirect contact, by droplets (particles ≥10 μm) and potentially through droplet nuclei (particles ≤5 μm) which may be inhaled into the lower airways.^3^, ^4^, ^5^, ^6^ Lower respiratory tract infection is associated with increased disease severity and mortality compared to infection of the upper respiratory tract.^7^, ^8^ Understanding determinants of influenza A virus (IAV) bioaerosol‐mediated transmission including environmental factors such as RH and temperature is relevant to mitigating spread.^9^, ^10^ RH can influence virion size, infectivity, and bioaerosol sampler performance. Early work with IAV determined that infectivity was best maintained at lower RH conditions.^11^ Noti et al also conducted experiments using simulated coughs and demonstrated that IAV aerosol infectivity was maximized at low RH.^12^ Furthermore, Lowen et al suggested that IAV transmission is highest at low RH, moderate at high RH (65%), and lowest at an intermediate RH (50%) using the guinea pig model.^10^ In addition, the range of RH may vary significantly depending on climate (temperate vs tropical) and setting (health care vs agricultural or wet markets). The overall effect of RH on IAV infectivity and transmission is still under investigation, but the effective performance of bioaerosol sampling devices at a range of RH is important for characterizing virus‐laden bioaerosols under different conditions.

An area of IAV research currently lacking attention is the effect of widespread non‐steroidal anti‐inflammatory drugs (NSAIDs) and other antipyretic use on IAV emission by infected hosts. Early experiments indicated that influenza virus–infected ferrets treated with antipyretic compounds experienced increased nasal shedding, potentially leading to increased risk of transmission.^13^ Mathematical modeling from Earn et al suggested that antipyretic use increases the infectious period and contact time with influenza virus–infected individuals, increasing the population‐level transmission risk.^14^ The ubiquitous use of NSAIDs among patients with influenza‐like symptoms underscores the importance of determining whether an association between NSAID use and emission of influenza virus into the air exists, and extension of host emission and sampling studies to determine whether NSAIDs could alter viral shedding represents important opportunities to fill this knowledge gap.

Assessing the burden of influenza virus in the air is technically and operationally challenging in real‐world settings such as healthcare institutions.^15^, ^16^, ^17^ Clinical and environmental studies have utilized a range of instruments to determine the risk of exposure to virus‐laden bioaerosols in health care and agriculture.^18^, ^19^, ^20^, ^21^, ^22^, ^23^, ^24^, ^25^, ^26^, ^27^, ^28^, ^29^, ^30^, ^31^, ^32^ Limited study sizes underscore the need for consistent approaches across studies in similar settings in order to generate robust comparative data to form clearer conclusions. Bioaerosol sampling devices are essential to the investigation and characterization of IAV bioaerosol emissions and transmission. Many sampling devices have been employed to collect a number of different pathogens but were not explicitly designed for the collection of viruses and preservation of viral infectivity. The selection of a suitable bioaerosol sampling device is challenging since collection efficiency is influenced by the pathogen in question, sampler flow rate, the environment, sampling time, and other technical and operational aspects of each device. Other factors such as cost and ease of use also influence device selection. Currently, there is no prevailing standard for the selection of a bioaerosol sampling device.

Filter‐based bioaerosol sampling devices, cyclone samplers, and cascade impactors are the most commonly used and accessible instruments for recovery and detection of viruses, though none were explicitly designed for this purpose.^17^, ^21^, ^22^, ^33^ Filter‐based instruments use membranes to collect particles as air is drawn through, whereas cyclone samplers recover particles by inertia, and some are capable of size fractionation.^15^, ^33^ Cascade impactors collect particles on solid or liquid media and may provide size fractionation as well.^15^, ^34^ These three portable sampler types have potential for implementation in standard practice for assessing the burden of virus in the air.

The primary objective of this study was to assess and compare bioaerosol sampling devices (Table 1) using (a) an in vitro, environmentally controlled artificial bioaerosol system at a range of different RH conditions and (b) an in vivo bioaerosol system utilizing influenza virus–infected ferrets under controlled environmental conditions. Secondarily, we also sought to examine the impact of NSAIDs on bioaerosol emission in influenza virus–infected ferrets to address its potential as a determinant of bioaerosol emission.

**Table 1:** Summary of characteristics, inclusion rationale, and recommendations for the polytetrafluoroethylene (PTFE) filter, NIOSH cyclone sampler, and Andersen impactor

Bioaerosol sampler	Characteristics	Rationale for inclusion	Recommendation	References
PTFE filter	Low‐volume air sampler Samples onto dry filter Disposable cassette Does not resolve particle size	Demonstrated efficiency in vitro and previously used in ferret model Compact, disposable, user‐friendly, commercially available, thus amenable for experimental and field use as well as multicenter studies	Clinical and environmental settings Ideal for RNA and infectivity detection Avoid areas with extremely high relative humidity	17, 18, 27, 28, 29
NIOSH cyclone sampler	Low‐volume air sampler Samples into dry tubes and filter Main instrument reusable (requires autoclaving) Able to differentiate particle size in three ranges	One of the more widely used instruments for the collection of viral bioaerosols in clinical settings and simulations Compact and portable Capable of size fractionation	Clinical and environmental settings Ideal for RNA and infectivity detection Decontamination necessary between uses	12, 16, 22, 30, 31
Andersen impactor	Mid‐volume air sampler Samples onto media Reusable (requires autoclaving) Able to differentiate particles size, number and ranges depend on the number of stages used	Used for viral bioaerosol collection in both healthcare and agricultural settings Capable of size fractionation	Difficult with liquid in field settings Ideal for RNA detection in controlled settings Avoid areas with high relative humidity	21, 32

## 2 MATERIALS AND METHODS

### 2.1 Ethics statement

Animal experiments were completed in a biosafety level 2+ containment facility at the Sunnybrook Research Institute (SRI, Toronto, Canada) in compliance with guidelines set by the Canadian Council on Animal Care and with approval of the SRI animal care committee.

### 2.2 Cells and viruses

Madin‐Darby canine kidney cells (MDCK cells, ATCC) were maintained in Eagle's minimum essential media (Wisent), 10% fetal bovine serum (FBS, Wisent), and penicillin (100 UI/mL)/streptomycin (100 μg/mL; P/S, Wisent). Influenza A/Ontario/2016 (H1N1)‐like and influenza A/Ontario/2016 (H3N2)‐like viruses were obtained from clinical samples at Sunnybrook Health Sciences Centre (Toronto, Canada) and subsequently passaged and plaque‐purified.^35^ These were used for artificial aerosolization experiments. Ferrets were inoculated with egg‐passaged influenza A/California/07/2009 virus (Cal/09).

### 2.3 Artificial aerosolizations

**Figure 1:**
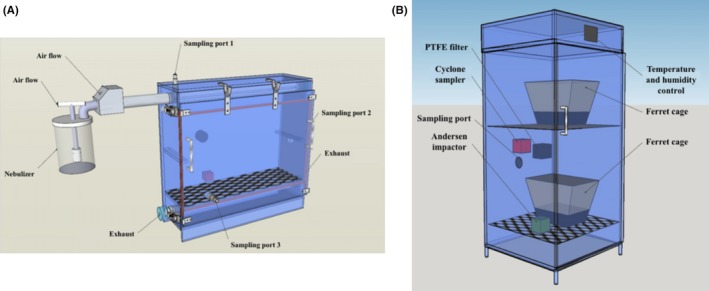
Artificial bioaerosol and environmental chambers. A, Custom‐built artificial aerosolization chamber with Collison nebulizer and B, Caron environmental test chamber for ferret experiments. PTFE filter (black), NIOSH cyclone sampler (red), and Andersen impactor (green) were used to sample influenza A virus–laden bioaerosols. All images were created or modified with SketchUp Pro software. Modified from Verreault, D

H1N1 (1.0 × 10^7^ plaque‐forming units (PFU)/ml) and H3N2 (1.28 × 10^6^ PFU/mL) influenza viruses were diluted in phosphate‐buffered saline (PBS, 1:5) and nebulized separately (BGI Collison Nebulizer, MesaLabs) into a custom‐built, pressurized, artificial aerosolization chamber (55.9 × 48.3 × 16.5 cm; Figure 1A) using HEPA‐filtered medical air at 6 L/min. The chamber was purged with sterile water preceding each 15‐minute virus nebulization. Sampling was conducted using a 1.0 μm pore size, 37‐mm polytetrafluoroethylene filter cassette (PTFE filter; SKC Limited), and the 2‐stage National Institute of Occupational Safety and Health cyclone sampler (NIOSH cyclone sampler; stage 1: collected particles >4.0 μm, stage 2: collected particles 1.0‐4.0 μm, NIOSH filter: collected particles <1 μm, filter is 3.0 μm pore size, 37 mm, PTFE) for 10 minutes at 3.5 L/min ± 5% (Gilian Air Sampling Pump, Sensidyne) starting 5 minutes after initiation of nebulization. The high flow rate of the 6‐stage viable Andersen cascade impactor (Andersen impactor; Tisch Environmental) precluded use with this system. RH and temperature were monitored throughout the experiments. RH within the chamber was maintained using a portable humidifier (Boneco), and temperature was maintained within a range of 19‐23°C. Three different RHs were tested, low (<25%), medium (47%‐53%), and high (78%‐83%; n = 3 for each). Sampling devices were placed inside the chamber, except for the PTFE filter at high RH due to filter saturation. Thus, sampling with the PTFE filter during high RH conditions was conducted from outside the chamber via connecting tubing. The chamber was cleaned with ethanol and purged with water for 25 minutes before each nebulization/sampling event.

### 2.4 Ferret experiments

Because ferret clinical features of disease are more similar to humans compared with other animal models including guinea pigs, they are well suited for the study of infectious bioaerosols responsible for transmission.^3^, ^36^, ^37^, ^38^, ^39^, ^40^ Six‐month‐old male influenza virus–free ferrets (Triple F farms) were screened for influenza A and B virus antibodies by hemagglutination inhibition, and mid‐turbinate swabs were tested for influenza virus RNA by PCR on arrival.^41^ Animals were housed in HEPA‐filtered ventilated isolators. Ferrets were anesthetized with isoflurane and inoculated intranasally with 1 mL Cal/09 at 10^6^ PFU diluted in PBS, then placed in a Caron 6030 environmental test chamber (environmental chamber; 58 × 65.5 × 75.7 cm; Figure 1B) at 20°C and 20% RH. For experiments using NSAIDs, animals were injected daily with PBS (untreated) or meloxicam (0.1‐0.2 mg/kg, treated) subcutaneously (n = 4 per group). The selection of meloxicam was based on its safety and tolerability in ferrets. Four ferrets were present in the environmental chamber and infected with influenza virus at a time. Rectal temperature and body weight were recorded, and animals were nasal‐washed with 2 mL of PBS under isoflurane anesthesia on days 1, 3, 5 and 7 post‐inoculation (p.i.) as previously described.^42^ Bioaerosol sampling of either untreated or treated groups of ferrets was conducted after the environmental chamber was undisturbed for a minimum of 6 hours. The PTFE filter and NIOSH cyclone sampler were used on days 1, 3, 5, and 7 p.i. at 3.5 L/min ± 5% for 2 hours, and the Andersen impactor was used on days 2, 4, 6, and 8 p.i. at 28.3 L/min for 30 minutes; petri dishes were filled with viral transport media (VTM; 1X P/S, 0.5% BSA, DMEM‐F12) to the appropriate height specified for proper size fractionation and placed in each stage of the Andersen impactor. The Andersen impactor was then placed at the bottom of the chamber during use. One sample was collected per instrument per group of ferrets (untreated or treated) at each time point. In a separate experiment, PTFE filter sampling of individual influenza virus–infected ferrets was subsequently conducted in the artificial aerosolization chamber (Figure 1A) under similar experimental conditions.

### 2.5 Sample processing and analysis

Bovine serum albumin (BSA, 0.5%) was added to nasal wash sample supernatants after centrifugation at 800 g for 5 minutes at 4°C. The PTFE filter was vortexed with 2 ml VTM for 1 minute, and the NIOSH cyclone sample tubes and filter were vortexed with 2, 0.5, or 1 mL of VTM for 1 minute for stages 1, 2, and the NIOSH filter, respectively. All samples were stored at −80°C after processing. Viral titers from nasal wash and bioaerosol samples (from bioaerosol sampling devices) were quantified by plaque assay in MDCK cells and reported as PFU. Viral RNA from air samples was extracted using the MagMAX™ Viral RNA Isolation Kit (Thermo Fisher Scientific) and stored at −80°C. Reverse transcriptase‐quantitative polymerase chain reaction (RT‐qPCR) of all air samples was conducted using primers targeting IAV matrix gene^41^ and the SuperScript™ III Platinum™ One‐Step qRT‐PCR Kit (Thermo Fisher Scientific).

### 2.6 Statistical analysis

All data were initially tested for normality using the D'Agostino‐Pearson omnibus normality test. Normally distributed data were tested using either an unpaired t test or one‐way ANOVA to determine statistical significance. Following ANOVA testing, Tukey's multiple comparisons test was utilized to determine groups that were statistically different. Non‐normally distributed data were assessed using either the Mann‐Whitney test or the Kruskal‐Wallis test to determine statistical significance (GraphPad Prism 6 software). The ratio of viral RNA to infectious virus was determined by dividing the viral RNA collected (copies) by infectious virus collected (PFU).

## 3 RESULTS

### 3.1 PTFE filter and NIOSH cyclone samplers collected IAV RNA and infectious virus from artificial aerosols under a range of RH

To evaluate the ability of the PTFE filter and NIOSH cyclone sampler to collect influenza virus RNA and infectious virus, we aerosolized H1N1 and H3N2 influenza viruses into a custom‐built artificial aerosolization chamber (Figure 1A). The Andersen impactor was not used in this setting because the flow rate was too high relative to the low volume of the chamber. The PTFE filter and NIOSH cyclone samplers collected similar quantities of H1N1 and H3N2 RNA and infectious virus under all RH conditions (Table 2). The ratio of viral RNA to infectious virus was determined to assess the loss of infectivity during the aerosolization, transit, and collection^17^ and this was similar to both H1N1 and H3N2 influenza viruses (Table 2).

**Table 2:** Viral RNA and infectious virus collected from the PTFE filter and NIOSH cyclone sampler after aerosolization of H1N1 and H3N2 influenza viruses, according to RH

Influenza virus	RH condition^a^	Viral RNA (copies/L air^b^ ± SEM^c^)		Infectious virus (PFU/L air^d^ ± SEM^c^)		Viral RNA to infectious virus ratio (copies/PFU)^e^	
		PTFE filter	NIOSH cyclone sampler	PTFE filter	NIOSH cyclone sampler	PTFE filter	NIOSH cyclone sampler
H1N1	Low	2.4 × 10^5^ ± 5.7 × 10^4^	2.4 × 10^5^ ± 6.4 × 10^4^	1.2 × 10^2^ ± 6.1 × 10^1^	1.3 × 10^2^ ± 7.4 × 10^1^	2.1 × 10^3^	1.9 × 10^3^
	Medium	3.0 × 10^5^ ± 9.1 × 10^4^	2.9 × 10^5^ ± 9.9 × 10^4^	1.7 × 10^1^ ± 1.6 × 10^1^	3.1 × 10^1^ ± 2.3 × 10^1^	1.8 × 10^4^	9.5 × 10^3^
	High	1.8 × 10^5^ ± 3.0 × 10^4^	1.9 × 10^5^ ± 6.4 × 10^4^	3.4 × 10^1^ ± 1.1 × 10^1^	5.4 × 10^1^ ± 2.6 × 10^1^	5.1 × 10^3^	3.4 × 10^3^
H3N2	Low	3.4 × 10^6^ ± 5.7 × 10^5^	3.5 × 10^6^ ± 3.3 × 10^5^	2.9 × 10^2^ ± 1.6 × 10^2^	3.3 × 10^2^ ± 1.9 × 10^2^	1.2 × 10^4^	1.1 × 10^4^
	Medium	1.9 × 10^6^ ± 6.2 × 10^5^	1.7 × 10^6^ ± 5.5 × 10^5^*	1.3 × 10^2^ ± 2.7 × 10^1^	1.5 × 10^2^ ± 2.8 × 10^1^	1.4 × 10^4^	1.1 × 10^4^
	High	1.8 × 10^6^ ± 2.5 × 10^5^	1.8 × 10^6^ ± 2.9 × 10^5^	1.8 × 10^2^ ± 2.8 × 10^1^	2.1 × 10^2^ ± 1.3 × 10^1^	1.0 × 10^4^	8.6 × 10^3^

a Low is <25% relative humidity, medium is 47%‐53% relative humidity, and high is 78%‐83% relative humidity.

b Viral RNA was determined by RT‐qPCR and presented as copies of RNA per liter air sampled.

c SEM = standard error of the mean (n = 3 per relative humidity condition).

d Infectious virus was determined by plaque assay and presented as PFU per liter air sampled.

e Viral RNA to infectious virus ratio was determined by dividing viral RNA copies per liter air sampled by PFU per liter air sampled.

* Significantly less H3N2 RNA recovered compared to the NIOSH cyclone sampler at low relative humidity (*P* < .05).

RH may affect viral viability in aerosols and bioaerosol sampler collection; thus, we also sought to compare instrument performance under a range of RH conditions. The RH within the artificial aerosolization chamber was maintained throughout aerosolizations at low (<25%), medium (47%‐53%), or high (78%‐83%) RH conditions. There was no significant difference between bioaerosol sampler collection at different RH conditions for RNA or infectious virus (*P* > .05, Table 1) except significantly less H3N2 RNA was collected by the NIOSH cyclone sampler at medium RH compared to low RH (*P* = .032; Table 2).

**Figure 2:**
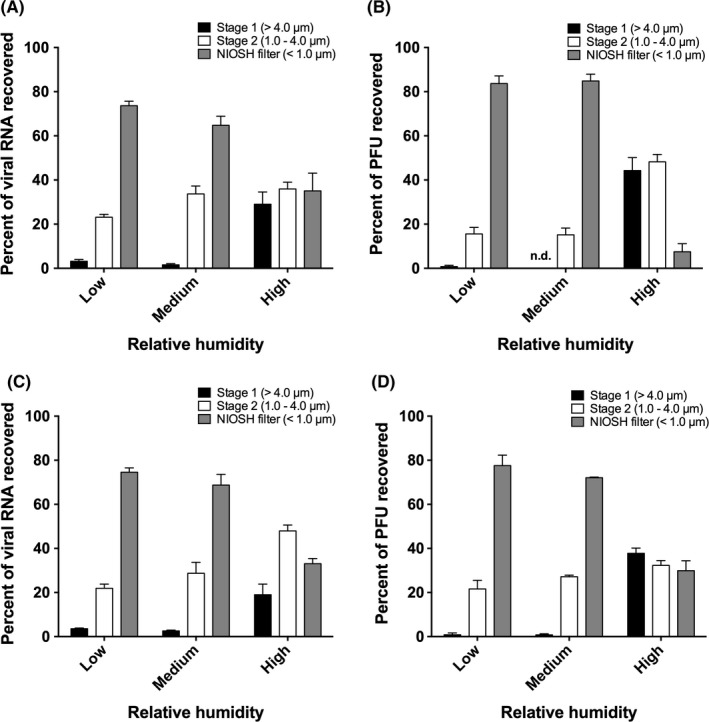
Size fractionation of influenza viral RNA and infectious virus collected by the NIOSH cyclone sampler. Predominately <1.0 μm particles formed at low and medium RH conditions. Viral RNA and infectious virus collected as A, percent of total H1N1 influenza virus RNA recovered, B, percent of total H1N1 influenza virus PFU recovered, C, percent of total H3N2 influenza virus RNA recovered, and D, percent of total H3N2 influenza virus PFU recovered by the NIOSH cyclone sampler according to particle size. Particle size ranges are >4.0 μm (black), 1.0‐4.0 μm (white), and < 1.0 μm (gray). Data are presented as means ± SEM, n = 3 for each relative humidity condition, n.d., not detected

Figure 2 indicates the size distribution of particles collected by the NIOSH cyclone sampler to provide further information on the influence of RH on influenza virus particle size. Similar particle sizes for both H1N1 and H3N2 influenza viruses were recovered under low and medium RH conditions using the NIOSH cyclone sampler, and the majority of infectious virus was recovered from the NIOSH filter (<1.0 μm) under these conditions (Figure 2). There were variable quantities of RNA and infectious virus from particles collected under high RH conditions.

### 3.2 Collection of bioaerosols emitted by IAV‐infected ferrets

**Figure 3:**
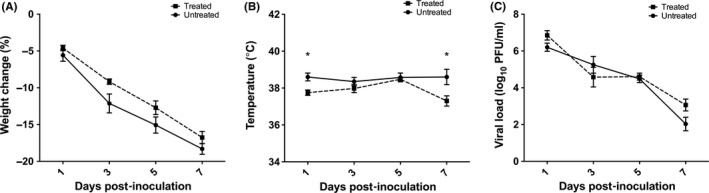
No difference was determined for weight change or viral load between untreated and treated ferrets. Ferrets treated with meloxicam had significantly lower rectal temperatures on days 1 and 7 p.i. A, Weight (percent change from baseline) and B, temperature (°C) were recorded on day 1 and alternating days p.i. C, Viral load (log_10_ PFU/mL) was determined from nasal washes on day 1 and alternating days p.i. Data are presented as means ± SEM, n = 4 ferrets per group. * *P* < .05

Next, we sought to collect IAV using bioaerosol samplers from influenza virus–infected ferrets. We also sought to determine whether NSAID administration affected influenza virus–laden bioaerosol production in a mammalian model. Weight loss was similar between untreated and NSAID (meloxicam)‐treated influenza virus–inoculated ferrets (Figure 3A). Treated ferrets had significantly lower temperatures on day 1 and 7 p.i. (Figure 3B, *P* = .016 and *P* = .041, respectively), but no fevers were noted. Nasal wash viral loads were highest on day 1 p.i., reaching 6.20 ± 0.22 log_10_ PFU/mL for untreated ferrets and 6.85 ± 0.26 log_10_ PFU/mL for treated ferrets, and declined until termination (Figure 3C) with no significant difference between untreated and treated animals (*P* > .05; Figure 3C).

**Figure 4:**
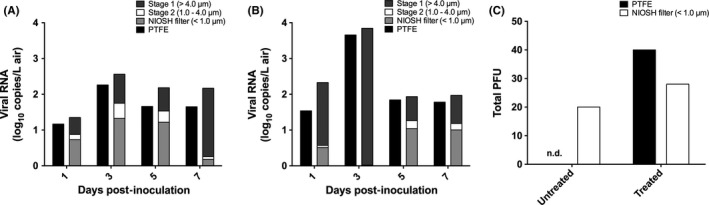
Polytetrafluoroethylene (PTFE) filter and NIOSH cyclone samplers retained viral RNA from the air within the environmental chamber used to house influenza virus–infected ferrets. A, Viral RNA (log_10_ copies/L air) collected from untreated ferrets by the PTFE filter (black) and NIOSH cyclone sampler (>4.0 μm = dark gray, 1.0‐4.0 μm = white, and <1.0 μm = light gray). B, Viral RNA (log_10_ copies/L air) collected from treated ferrets by the PTFE filter (black) and NIOSH cyclone sampler (>4.0 μm = dark gray, 1.0‐4.0 μm = white, and <1.0 μm = light gray). C, Infectious virus (total PFU) collected by the PTFE filter (black) and NIOSH cyclone sampler stage 3 (<1.0 μm, white) on day 3 p.i. One sample collected per day for each sampler (4 ferrets per sample), n.d., not detected

The PTFE filter and NIOSH cyclone sampler collected the most viral RNA on day 3 p.i. for both untreated and treated ferrets (Figure 4A, 4B). PTFE filters collected 2.26 log_10_ copies/L air for untreated and 3.66 log_10_ copies/L air for treated animals, while the NIOSH cyclone sampler collected 2.56 log_10_ copies/L air for untreated and 3.84 log_10_ copies/L air for treated ferrets on day 3 p.i. (Figure 4A, 4B). More viral RNA was collected from the air of treated ferrets than untreated ferrets, though statistical significance could not be determined due to small sample size (Figure 4A, 4B). Infectious virus was collected on day 3 p.i. from the PTFE filter sampling treated ferrets and from the NIOSH cyclone sampler sampling both untreated and treated ferrets (Figure 4C).

**Figure 5:**
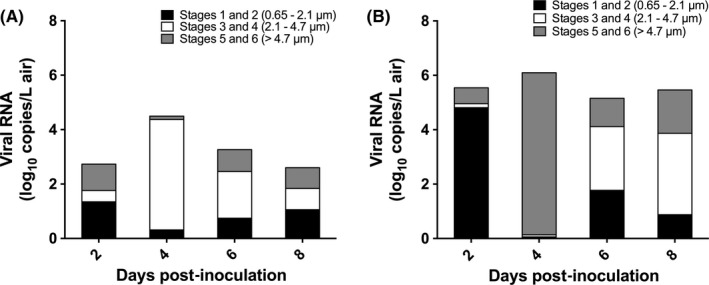
Viral RNA collected from the air emitted by IAV‐infected ferrets using the Andersen impactor. A, Viral RNA (log_10_ copies/L air) collected from untreated ferrets by the Andersen impactor (>4.7 μm = gray, 2.1‐4.7 μm = white, and 0.65‐2.1 μm = black). B, Viral RNA (log_10_ copies/L air) collected from treated ferrets by the Andersen impactor (>4.7 μm = gray, 2.1‐4.7 μm = white, and 0.65‐2.1 μm = black). One sample collected per day for each sampler (4 ferrets per sample)

The Andersen impactor collected the most viral RNA on day 4 p.i. for both untreated and treated ferrets. Andersen impactor stages were combined to reflect upper (>4.7 μm), mid (2.1‐4.7 μm), and lower (0.65‐2.1 μm) airway distributions in the respiratory tract^43^; 4.49 log_10_ copies/L air were collected from untreated ferrets, and 6.09 log_10_ copies/L air were collected from treated ferrets (Figure 5A, 5B). More viral RNA was collected by the Andersen impactor from ferrets treated with meloxicam than untreated ferrets, but statistical significance could not be determined (Figure 5A, 5B).

We also inoculated ferrets from untreated and meloxicam‐treated groups and individually sampled each animal by nasal washing and using the PTFE filter (Figure S1). Approximately 10.4% of aerosol samples were positive for IAV RNA and only 3 of 48 aerosol samples were positive for infectious IAV (Figure S2). We attempted to determine a relationship between viral load and virus (RNA and infectious virus) collected from the air using a Fisher's exact test, chi‐squared test, and a Spearman correlation but did not find a significant difference.

## 4 DISCUSSION

The merits of bioaerosol sampling for respiratory viruses are coming to the forefront as researchers have used this approach to detect virus from air emitted by individuals with laboratory‐confirmed influenza,^21^, ^22^, ^44^ during severe acute respiratory syndrome coronavirus (SARS‐CoV) and Middle East respiratory syndrome coronavirus (MERS‐CoV) outbreaks,^45^, ^46^ and to assess the environmental burden of avian influenza viruses in wet markets.^47^, ^48^ In this study, we evaluated three portable samplers using artificial aerosols and a translational, in vivo model. Table 1 outlines a summary of characteristics, rationale, and recommendations for each device. The PTFE filter and NIOSH cyclone sampler recovered RNA and infectious H1N1 and H3N2 influenza viruses under different RH conditions and from influenza virus–infected ferrets. This evidence combined with their cost‐effective nature and user‐friendly design makes the PTFE filter and NIOSH cyclone sampler good candidates for the detection of influenza virus–laden bioaerosols in multiple settings. The Andersen impactor collected RNA from influenza virus–infected ferrets and is a good candidate for experiments in controlled settings since it is less maneuverable relative to the PTFE filter and NIOSH cyclone samplers.

The ratio of viral RNA to infectious virus is important for understanding the burden of influenza virus in the air and an indication of the influence of aerosolization, transit, and collection on the infectivity of the virus.^17^ In this study, the average ratio of viral RNA to infectious virus was 1.02 × 10^4^ copies/PFU for the PTFE filter and 7.55 × 10^3^ copies/PFU for the NIOSH cyclone sampler. The ratios of collected viral RNA to infectious virus for both the PTFE filter and NIOSH cyclone sampler are slightly higher than the ratio for the nebulizer (3.66 × 10^3^ copies/PFU), indicating a potential loss of infectivity during the aerosolization and collection process or due to other factors that may disrupt the viral envelope or otherwise affect infectivity. Despite this potential loss, both bioaerosol samplers recorded similar ratios, indicating their comparable ability to collect infectious virus. Both samplers were also able to collect as much as 10^4^ PFU of virus. Previous work has suggested that dry sampling or sampling onto a dry surface such as a filter may limit the ability of bioaerosol samplers to collect infectious virus,^17^ but our work indicates that under certain environmental conditions, it is possible to do so with dry or filter sampling, despite loss of infectivity due to aerosolization and/or sampling processes.

IAV transmission is potentially enhanced at low temperature and low RH, while the reciprocal effect is observed when temperature and humidity increase.^10^, ^49^, ^50^ Under certain conditions, temperature and humidity may have limited or variable effects on transmission between animals.^11^, ^51^, ^52^, ^53^ Utilizing an established guinea pig model of transmission,^54^ Lowen et al demonstrated that low RH (20%‐30%) is optimal for influenza virus transmission.^55^ Prussin et al also demonstrated the same influence of low RH on infectivity using bacteriophages.^50^ Testing bioaerosol samplers under a range of IAV transmission conditions is essential for determining the usefulness of these sampling devices. Our data show RH had some effect on the ability of the PTFE filter and NIOSH cyclone sampler to detect viral RNA and infectious virus. A significant decrease in H3N2 influenza virus RNA collected by the NIOSH cyclone sampler at medium RH is in keeping with the results shown by Lowen et al and further demonstrates the influence of increasing RH on the collection of IAV from bioaerosols. The PTFE filter collected virus well under low and medium RH conditions but became saturated with liquid under high RH, representing a potential limitation. The NIOSH cyclone sampler performed similarly, but saturation was not a factor at high RH; viral RNA and infectious virus were collected across all RH conditions. High RHs can inhibit bioaerosol sampling devices through saturation with liquid, potentially affecting the ability to sample in tropical climates or humid environments such as sometimes seen in swine barns or poultry markets. Furthermore, there were variable quantities of viral RNA and infectious virus from particles collected by the NIOSH cyclone sampler during high RH conditions, potentially reflecting enhanced but varying degrees of virus aggregation and highlighting the difficulty of recovering material under these conditions. RH may influence both the characteristics of aerosolized viral particles as well as sampler performance, and unfortunately, we cannot be certain what the relative contribution of each potential factor is to the net collection of virus using an in vitro experimental system. The effect of RH on infectivity was further demonstrated in a study looking at IAV droplets in various saline solutions. The authors found that viability was lowest at 50%‐99% RH and suspected these conditions led to ongoing evaporation, which increased salt concentrations to levels that are potentially harmful to the virus.^56^ In addition, the different effects of RH on H3N2 and H1N1 subtypes could not be adequately explored with the static system we employed, although others have recently done so.^56^

The use of ferrets to test IAV bioaerosol collection using three bioaerosol sampling devices is an excellent method for understanding the performance of these devices, though not all samplers could be tested concomitantly due to operational and special confines of the housing system required to maintain chamber equilibrium and temperature and RH constant. This environment is a controlled setting intended to reflect collection from emitting hosts. We found that the PTFE filter and NIOSH cyclone sampler were capable of collecting infectious virus from both groups of influenza virus–infected ferrets on day 3 p.i. The Andersen impactor collected impressive amounts of viral RNA, but no infectious virus was detected. This instrument collects at a higher flow rate (28.3 L/min) and has the ability to size fractionate particles, which are enticing attributes when choosing a bioaerosol sampler. However, the difficulties with maneuvering the apparatus with liquid media while inside the environmental chamber make it vulnerable to pump damage. The Andersen impactor is therefore best suited for controlled, experimental settings. Contrary to the Andersen impactor, the PTFE filter and NIOSH cyclone samplers were easy to manipulate inside the environmental chamber (compact, lightweight, few parts). These devices can be fixed to walls for static sampling or worn by personnel for personal breathing zone sampling. These attributes combined with the ability to collect viral RNA, and infectious virus makes these samplers more feasible for assessing the burden of influenza virus in the air in various settings, and more conducive for use in multicenter studies.

It has been suggested that anti‐inflammatory NSAIDs such as meloxicam^57^ may be associated with increased viral shedding of influenza virus; mathematical and experimental work suggest that suppression of fever can lead to increased influenza virus replication in the upper respiratory tract and consequently increased transmission.^13^, ^14^, ^58^ We leveraged these bioaerosol experiments to also investigate whether treatment with an NSAID affected bioaerosol emissions. Following inoculation with influenza virus, ferrets experienced significant weight loss and typical shedding patterns. Although statistical significance could not be determined, all three bioaerosol samplers captured higher levels of viral RNA and infectious virus from the meloxicam‐treated group compared to their untreated counterparts. This indicates a potential enhancement of influenza virus bioaerosol production after NSAID treatment through an unknown mechanism, and could play a significant role during influenza epidemics and pandemics due to the widespread use of NSAIDs such as ibuprofen.^59^ Transmission was not tested in this study since this would have halved the number of inoculated animals and significantly increased the variability in recovery of viral RNA and infectious virus, further limiting statistical analysis. Extensive work is still needed to better understand the relationship between NSAIDs and influenza virus replication and transmission to determine whether their use actually contributes to the spread of influenza virus.

There were several limitations in this study, including the choice of bioaerosol sampling devices; glass liquid impingers were not included since their use in a clinical environment would be prohibited. The use of liquid media instead of solid agar in the plates of the Andersen impactor may also be problematic since the volume of liquid or agar present in each plate is critical for proper size fractionation of particles and the potential loss of liquid during the collection process may lead to a misrepresentation of particle size distribution. Kormuth et al recently demonstrated the limited influence of RH on the infectivity of physiologically relevant fine aerosols and droplets,^60^ and in our study, virus for aerosolization was diluted in saline lacking physiological components of the extracellular matrix. Also, the evaluation of these samplers did not include characterization of precise performance parameters of each device, including limit of collection. An ultraviolet aerodynamic particle sizer (UV‐APS) would be required to accurately determine sampling efficiency. Finally, the small sample sizes of animal experiments, even if in keeping with most ferret studies, present difficulties when attempting to draw firm, statistically significant conclusions.

## 5 CONCLUSION

In summary, the Andersen impactor has a high flow rate, the ability to size fractionate particles, and is commercially available. The cumbersome nature and cost of the Andersen impactor impacts the feasibility for widespread clinical and field use, but this instrument may be ideal for collection of IAV RNA in experimental and other well‐controlled settings. The NIOSH cyclone sampler is portable, lightweight, is able to size fractionate bioaerosols, collects IAV RNA and infectious virus, and works well under multiple RH conditions. It is not commercially available and requires time for processing and decontamination but can be used for personal sampling and whenever particle size fractionation is desired in clinical and environmental settings. The PTFE filter is portable, lightweight, disposable, and easily manipulated, and can collect IAV RNA and infectious virus with minimal processing. The PTFE filter is extremely versatile; it can be used in clinical and environmental settings, for both personal sampling and static sampling. The PTFE filter, NIOSH cyclone sampler, and Andersen impactor are all viable options for bioaerosol sampling of IAV, depending on the application, and may be considered for field, clinical, or experimental studies. Determining which bioaerosol sampler to use has been historically difficult, but moving forward, this study provides valuable evidence to guide bioaerosol sampler selection.
