# Supplementary figures and images for: Evaluation of bioaerosol samplers for the detection and quantification of influenza virus from artificial aerosols and influenza virus–infected ferrets
Source: Influenza Other Respir Viruses. 2019 Sep 21;13(6):564–73. doi: 10.1111/irv.12678 (PMC6800310; doi:10.1111/irv.12678)

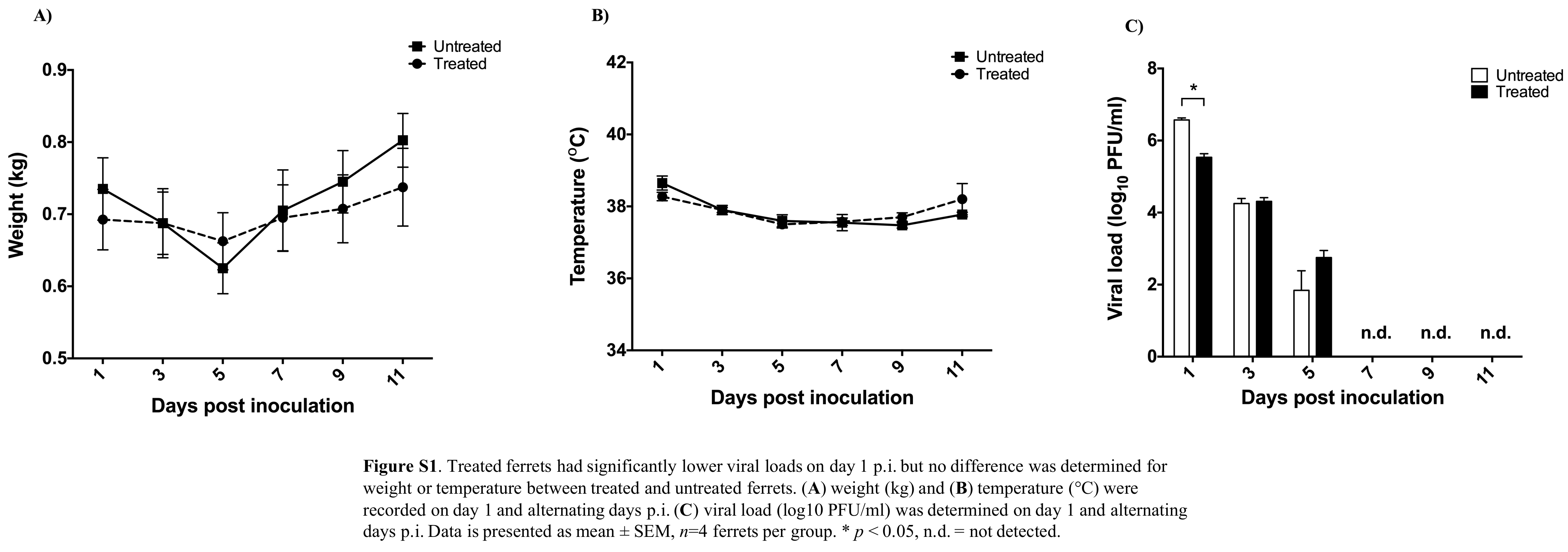

Supplement: Supplementary file 1 [file IRV-13-564-s001.tiff]

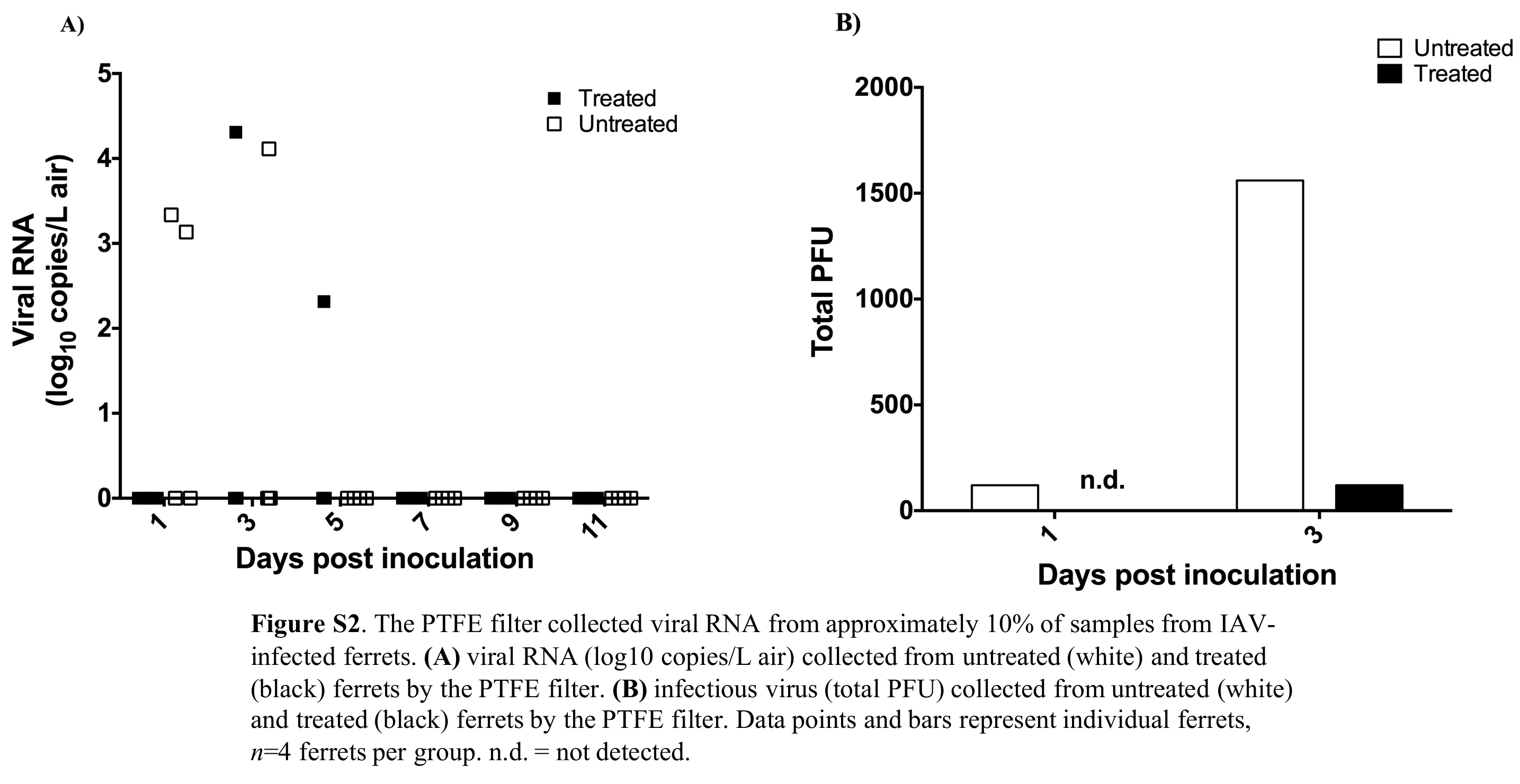

Supplement: Supplementary file 2 [file IRV-13-564-s002.tiff]
